# Serum Markers of Blood-Brain Barrier Remodeling and Fibrosis as Predictors of Etiology and Clinicoradiologic Outcome in Intracerebral Hemorrhage

**DOI:** 10.3389/fneur.2018.00746

**Published:** 2018-09-07

**Authors:** Matthew D. Howe, Liang Zhu, Lauren H. Sansing, Nicole R. Gonzales, Louise D. McCullough, Nancy J. Edwards

**Affiliations:** ^1^Department of Neurology, McGovern Medical School, University of Texas Health Science Center, Houston, TX, United States; ^2^Department of Neurology, Yale University School of Medicine, New Haven, CT, United States; ^3^Neuroscience Department, Kaiser Permanente, Redwood City, CA, United States

**Keywords:** intracerebral hemorrhage, biomarker, matrix metalloproteinase, fibronectin, TGF beta, cerebral amyloid angiopathy

## Abstract

**Background:** Intracerebral hemorrhage (ICH) is a stroke subtype associated with high disability and mortality. There is a clinical need for blood-based biomarkers that can aid in diagnosis, risk stratification, and prognostication. Given their role in the pathophysiology of ICH, we hypothesized markers of blood-brain barrier disruption and fibrosis would associate with neurologic deterioration and/or long-term functional outcomes. We also hypothesized these markers may be unique in patients with ICH due to cerebral amyloid angiopathy (CAA) vs. other etiologies.

**Methods:** Seventy-nine patients enrolled in prospective ICH registries at two separate hospitals (the University of Texas Health Science Center at Houston and Hartford Hospital) were included in this study. We assessed initial injury severity and admission variables along with measures of inpatient deterioration (hematoma expansion, perihematomal edema (PHE), and early and delayed neurologic deterioration) and functional outcome [modified Rankin Scale (mRS) score at discharge and 90 days]. Serial biospecimens were obtained at 5 pre-specified timepoints (within 24 h, 1–2, 3–5, 6–8, and 10 days); serum samples were analyzed for fibronectin, all three TGF-β isoforms, and 7 matrix metalloproteinases (MMPs).

**Results:** In our initial correlation analysis, MMP 10 and 3 were associated with hematoma expansion and early neurologic deterioration, whereas MMP 8 and MMP 1 were associated with PHE and delayed neurologic deterioration (respectively). Subacute levels of MMP 8 (sampled from day 6–10) positively correlated with PHE even after adjusting for multiple comparisons (*p* = 0.02). Acute levels of MMP 1, TGF-β1, and TGF-β3 were predictive of functional outcome, with TGF-β1 and TGF-β3 associating with 90 day mRS independent of age, hematoma volume, hemorrhage location, GCS, and IVH [*p* = 0.02; OR 1.03 (95% CI 1.0–1.05); *p* = 0.03; OR 3.1 (95% CI 1.1–8.8)]. When evaluated together as a panel, the cytokines distinguished patients with ICH due to CAA vs. ICH due to hypertension (AUC 0.81).

**Conclusions:** Serum levels of fibronectin, TGF-β, and MMPs may be useful in refining ICH etiology and prognosis. Further large-scale studies are needed to confirm these findings, particularly regarding patients with CAA.

## Introduction

Intracerebral hemorrhage (ICH) accounts for approximately 15% of strokes and is a major cause of morbidity and mortality worldwide, affecting approximately 2 million individuals each year (1). Nearly 40% of ICH patients will die within 30 days, and of survivors, functional independence within 6 months is achieved by only 20% (2, 3). For spontaneous ICH, hypertension remains the most frequent etiology, though certain subsets of patients (such as elderly patients) may have other ICH causes such as antithrombotic use or cerebral amyloid angiopathy [CAA; (3, 4)]. In ICH, the exposure of brain tissue to blood components results in various neuroinflammatory cascades that lead to secondary injury after ICH. For instance, thrombin induces the activation of matrix metalloproteases (MMPs) that can then degrade basement membrane components of the blood-brain barrier (BBB), such as the protein fibronectin (5–9). BBB disruption can result in an influx of peripheral immune cells into the brain along with the formation of cerebral edema, referred to as perihematomal edema (PHE). Other mediators, such as transforming growth factor-β (TGF-β), may instead regulate neuroinflammation and promote repair processes such as the formation of glial scars (10, 11).

While these ICH injury and repair processes have been studied to a far greater extent in experimental models of ICH, there is less literature on the role of these mediators in patients with ICH. Several studies have suggested MMPs, fibronectin, and/or TGF-β are elaborated in both the brains and peripheral blood of ICH patients, but it is unclear if serum levels of these mediators could serve as diagnostic or prognostic markers. For instance, TGF-β1 can induce amyloid deposition in cerebral blood vessels but as of yet, peripheral levels of TGF-β have not reliably distinguished CAA hemorrhage patients from other ICH patients (12, 13). In addition, the identification of prognostic serum markers could guide individualized patient care and rehabilitation strategies and hopefully aid in the improvement of ICH outcomes.

In this study, we performed an exploratory analysis of serum levels of MMPs, fibronectin, and TGF-β across time in patients with ICH in the hope of ascertaining if and when these cytokines were predictive of clinically relevant short- and long-term outcomes. We also examined whether these cytokines could be useful (either individually or as a grouped panel) as diagnostic of ICH etiology, specifically ICH due to CAA.

## Materials and methods

### Study population

For this study, we used ICH patient data and serum samples from the University of Texas Health Science Center at Houston (UTH) and Hartford Hospital (HH). The study was approved by the Institutional Review Boards at both institutions, and all patients (or their surrogates) were consented. We abstracted data from prospective ICH databases detailing admission data, radiologic findings, hospital course, and functional outcomes. Seventy-nine patients with spontaneous, supratentorial cerebral hemorrhages of moderate size were included in the study. All patients were enrolled within 24 h of ICH ictus. Patient exclusions were the following: underlying vascular lesion (aneurysm, arteriovenous malformation), traumatic brain injury, venous sinus thrombosis, infratentorial hemorrhage location, and/or inadequate clinical follow-up.

### Clinical and radiologic assessments

ICH severity was assessed via the National Institutes of Health Stroke Scale (NIHSS), Glasgow Coma Scale (GCS), and ICH score (14–16). Functional outcomes were assessed at discharge and 90 days post-injury by the modified Rankin Scale [mRS, (17)]. Our primary radiologic outcomes were hematoma volume (HV) and perihematomal edema (PHE), as quantified via computer-based analysis with MIPAV (Medical Image Processing, Analysis, and Visualization) software. Serial volumes were obtained by review of every head CT obtained during the patient's admission as a part of routine clinical care. ICH etiology was determined by the clinical team with independent verification by an experienced neurologist (NJE); patients were subdivided into deep hypertensive ICH, lobar ICH due to probable CAA (as defined via the modified Boston criteria), and lobar ICH due to “other” causes, such as coagulopathy (18). In the UTH cohort, the majority of CAA ICH patients had probable CAA with supporting pathological evidence.

### Serum sample collection

Serum samples were obtained from our study cohort serially and at pre-specified timepoints: timepoint 1 (within 24 h), timepoint 2 (1–2 days post-injury), timepoint 3 (3–5 days), timepoint 4 (6–8 days), and timepoint 5 (10 days). All biospecimens were sampled by a team blinded to the clinical status of the patient. Samples were processed within 1 h of collection and stored at−80 degrees Celsius.

### Measurement of serum analytes

Samples were thawed on ice for 1 h and thoroughly vortexed prior to beginning any assays. Serum fibronectin was measured using the Human Fibronectin Quantikine ELISA Kit (R&D Systems). Latent and active TGF-β1, TGF-β2, and TGF-β3 were measured using the Bio-Plex Pro TGF-β 3-PLEX assay (Bio-Rad). Similarly, MMP 1, MMP 2, MMP 3, MMP 7, MMP 8, MMP 9, and MMP 10 were measured using the Bio-Plex Pro Human MMP Panel (Bio-Rad). All assays were performed and read according to the manufacturer's instructions.

### Statistical analysis

Descriptive statistics were provided for demographic and baseline variables in our ICH patients. Nonparametric analysis (Spearman correlation, Wilcoxon rank sum test) was used to define serum cytokine associations with our outcomes of interest (hematoma expansion, early and delayed neurologic deterioration, PHE, discharge and 90 day mRS). For patients with serial samples (*n* = 36), we also grouped cytokine levels across acute (0–5 days) and subacute (6–10 days) timepoints, adjusting for multiple comparisons using the Benjamini and Hochberg procedure to control the false discovery rate (FDR) at 0.05 (19). Further analysis of cytokine association with functional outcome was performed via a multivariable logistic regression analysis adjusting for age, hematoma volume, hemorrhage location, admission GCS, and IVH. Logistic regression analysis was also used to generate a receiver operating characteristic (ROC) curve comparing cytokine signatures in CAA ICH patients vs. deep hypertensive ICH patients, with adjustment for hematoma volume. All data analyses were performed in SAS software 9.4 (Cary, NC).

## Results

### Baseline cohort findings

Baseline clinical variables are outlined in Table 1 (*n* = 79). The mean age of our cohort was 66.8 years old (± 14.3 years) and 39.7% of patients were female. Mean peak hematoma volume was 40.8 cm^3^ (± 26 cm^3^) and mean peak PHE volume was 26.5 cm^3^ (± 17.9 cm^3^). Intraventricular extension of hemorrhage was present in 65% of patients. 42 patients had deep hypertensive hemorrhages, and 37 had lobar hemorrhages.

**Table 1:** Baseline patient demographics and ICH variables.

**Variable**	**Total cohort (*n* = 79)**
Age, years	66.8 (± 14.3)
Female, n	27
Black, n	15
White, n	47
Admission GCS	13
Admission NIHSS	14
ICH score	2
Admission SBP, mm Hg	187.7
Admission DBP, mm Hg	106.5
Deep location, n	42
IVH, %	65.2%
Hematoma expansion, %	29.4%
Peak hematoma volume, cm^3^	40.8 (± 26)
Peak perihematomal edema, cm^3^	26.5 (± 17.9)
END, %	40%
DND, %	20%
mRS @ discharge	4
mRS @ 90 days	4

*GCS, Glasgow Coma Scale; NIHSS, National Institutes of Health Stroke Scale; ICH, intracranial hemorrhage; SBP, systolic blood pressure; DBP, diastolic blood pressure; IVH, intraventricular hemorrhage; END, early neurologic deterioration; DND, delayed neurologic deterioration; mRS, modified Rankin Scale*.

*Mean: age, admission SBP, admission DBP, peak hematoma volume, peak perihematomal edema*.

*Median: admission GCS, admission NIHSS, ICH score, mRS @ discharge, mRS @ 90 days*.

### Temporal association of serum markers with clinical deterioration and functional outcome

**Figure 1:**
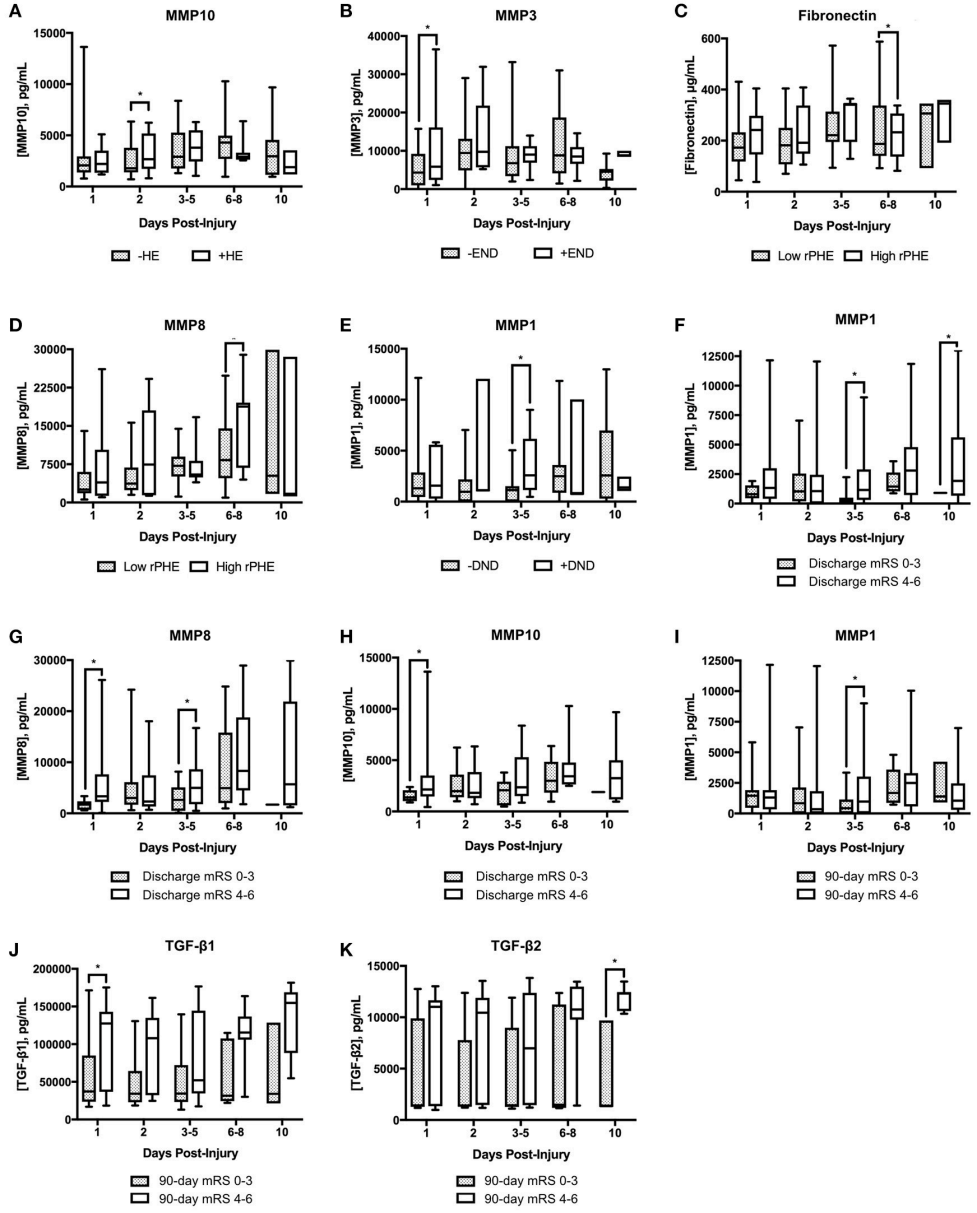
Box plots of serum cytokine levels across timepoints as separated by ICH outcomes. **(A)** MMP10 levels in patients without or with hematoma expansion (HE); **(B)** MMP3 in patients without or with early neurologic deterioration (END); **(C)** fibronectin in patients with low or high relative perihematomal edema (rPHE); **(D)** MMP8 levels in patients with low or high rPHE; **(E)** MMP1 in patients without or with delayed neurologic deterioration (DND); **(F–H)** cytokine levels in patients with favorable (0–3) vs. unfavorable (4–6) modified Rankin Scale (mRS) score at discharge; **(I–K)** cytokine levels in patients with favorable versus unfavorable mRS at 90 days. ^*^p ≤ 0.05. MMP, matrix metalloproteinase; TGF, transforming growth factor.

We characterized serum cytokine levels in our ICH patients across our 5 prespecified timepoints. 36 of our 79 patients had serial samples throughout. Univariate analysis was performed to ascertain cytokine associations with clinically relevant outcomes—hematoma expansion, early neurologic deterioration (END), delayed neurologic deterioration (DND), perihematomal edema volume, discharge mRS, and 90 day mRS. In Figure 1, we have displayed boxplots of serum cytokine levels across time in patients with or without our outcomes of interest; statistically significant associations are highlighted in Table 2. MMP 10 at an early timepoint (within 2 days of ICH ictus) was the only variable significantly associated with hematoma expansion (*p* = 0.03) and MMP 3 at timepoint 1 was the only variable significantly associated with END (*p* = 0.05). Interestingly, the “delayed” clinical deterioration variables—PHE and DND—were only associated with markers at later timepoints (MMP 8 and fibronectin at day 6–8 for PHE, *p* = 0.005 and 0.006; MMP 1 at day 3–5 for DND, *p* = 0.02). Serum markers associated with discharge disposition and mRS at discharge included MMP 1, MMP 8, and MMP 10. MMP 1 (*p* = 0.01), TGF-β1 (*p* = 0.05), and TGF-β2 (0.02) were associated with mRS at 90 days.

**Table 2:** Univariate analysis of serum marker associations with clinical and/or radiologic outcome measures.

**Variable**	**Serum cytokine**	**Timepoint (days post-injury)**	***P*-value**
HE	MMP 10	2	0.03
END	MMP 3	1	0.05
PHE	MMP 8	6–8	0.005
	Fibronectin	6–8	0.006
DND	MMP 1	3–5	0.02
mRS @ Discharge	MMP 8 MMP 10 MMP 1	1; 3–5 1 3–5; 10	0.02; 0.04 0.04 0.01; 0.02
mRS @ 90 days	TGF-β1 MMP 1 TGF-β2	1 3–5 10	0.05 0.01 0.02

*HE, hematoma expansion; PHE, perihematomal edema; MMP, matrix metalloproteinase; TGF, transforming growth factor*.

### Acute vs. subacute groupings

To determine if any of these markers would be useful if obtained within a temporal window rather than at one specific timepoint, we grouped acute (day 0–5) and subacute (day 6–10) timepoints together and reassessed the above associations. In this analysis, we corrected for multiple comparisons as described in the Methods section. Regarding our measures of short-term deterioration (hematoma expansion, END, DND, and PHE), the only association that remained significant upon adjustment was the association of subacute MMP 8 with PHE (*p* = 0.02). Functional outcomes at both discharge and at 90 days were significantly associated with acute levels of MMP 1 (*p* = 0.04, *p* = 0.007), TGF-β1 (*p* = 0.04, *p* = 0.003), and TGF-β3 (*p* = 0.04, *p* = 0.004). And, as displayed in Table 3, higher levels of TGF-β1 and TGF-β3 were predictive of poorer long-term functional outcome independent of patient age, hematoma volume, hemorrhage location, admission GCS, and IVH [*p* = 0.02, OR 1.03 (95% CI 1–1.05) and *p* = 0.03, OR 3.1 (95% CI 1.1–8.8), respectively].

**Table 3:** Multivariate logistic regression analysis of marker association with poorer long-term functional outcome.

**Serum cytokine**	Adjusted *p*-value^*^	**Odds ratio (95% CI)**
**TGF-**β**1**	**0.02**	**1.03 (1–1.05)**
TGF-β2	0.08	1.2 (0.9–1.5)
**TGF-**β**3**	**0.03**	**3.1 (1.1–8.8)**
MMP 1	0.3	1.5 (0.6–3.7)

CI, confidence interval.

* *Adjusted for patient age, hematoma volume, hemorrhage location, admission GCS, and IVH. Bold values are statistically significant (adjusted p < 0.05)*.

### Molecular signature associated with CAA hemorrhages

**Figure 2:**
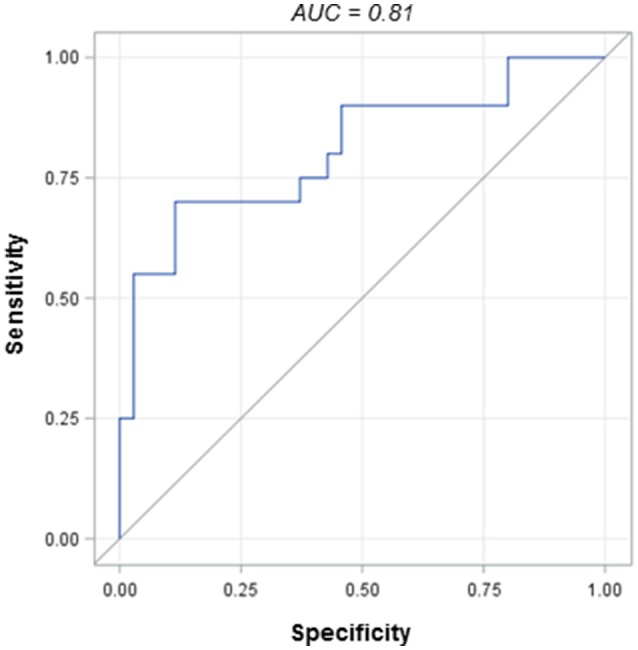
Receiver operating characteristic (ROC) curve for the cytokine panel in CAA vs. hypertensive ICH patients.

We next sought to determine if ICH etiology (ICH due to CAA, in particular) was associated with any particular serum marker or network of markers. When each marker was analyzed independently (adjusting for multiple comparisons), we did not find any statistically significant associations with ICH etiology. However, when we examined MMPs, fibronectin, and TGF-β together, the cytokine panel as a whole was able to distinguish CAA ICH patients from hypertensive ICH patients. Figure 2 demonstrates the receiver operating characteristic (ROC) curve, adjusted for hematoma volume, with an area under the curve (AUC) of 0.81.

## Discussion

The main findings of this study include the association of several of our candidate serum markers with various measures of ICH severity, deterioration, and functional outcome along with the identification of a serum molecular signature for patients with ICH due to CAA as compared to patients with ICH due to hypertensive hemorrhage. Several of the statistically significant associations presented here are novel, such as the association of MMP 8 with PHE. In addition, our study demonstrates certain associations being distributed throughout time, suggesting the time course of biosampling is relevant (the first several days vs. the first week or beyond). And though several of these markers have been previously demonstrated in other studies to be prognostic, this is the first study (to our knowledge) where these markers, when examined as a network, were useful as diagnostic markers for CAA-related ICH.

Numerous studies of ICH in both preclinical models and in patients have suggested a role for MMPs in the pathogenesis of ICH. For instance, in patients with ICH, MMP 3 and MMP 9 have been associated with deterioration due to cerebral edema and poorer functional outcomes (7, 8, 20, 21). In our study, MMP 3 and 9 were primarily associated with initial injury severity, hematoma volume, and early neurologic deterioration. We did not find a statistically significant association of MMP 3 or MMP 9 with PHE or long-term functional outcome. This may have been due to (1) the larger panel of markers being tested, and (2) our search was for outcome associations independent of previously demonstrated clinical prognosticators. For instance, in the study by Li et al, though initial analyses suggested MMP 3 was associated with absolute PHE volume, when a multivariable model including hematoma volume was used, MMP 3 was no longer an independent predictor of PHE—only HV was (20).

In our study, TGF-β1 and TGF-β3 were independently predictive of 90 day mRS, suggesting serum evaluations of these markers could be prognostically additive to scoring systems such as the ICH score. Taylor et al also reported an independent association of TGF-β1 with functional outcome in patients with ICH (10). In this study, though, rebounds in TGF-β1 levels by 72 h correlated with favorable functional outcome whereas in our study, higher absolute levels of TGF-β1/TGF-β3 throughout our sampling period correlated with poorer outcome. There are several potential explanations for the divergent findings: in the previous study, TGF-β1 was measured in plasma whereas we measured our cytokines in the serum; we did not compare cytokine levels to those of non-neurologic controls (whereas the previous study did); our patients were “sicker” overall—with higher hematoma volumes and ICH scores; we sampled additional timepoints through day 10. Interestingly, in our univariate analysis, higher TGF-β2 at day 10 in particular was associated with unfavorable 90 day mRS. Numerous studies of neuroinflammation in ICH have revealed a bitemporal time course where initially damaging cytokines may become helpful during subacute/chronic repair processes, and vice versa (22–25). Prolonged elevations of TGF-β peripherally may signal prolonged injury in the central nervous system; as a result, prolonged elevations of serum TGF-β may correlate with poorer recovery. Also, in preclinical models of stroke, absolute levels of TGF-β signaling was significantly higher in aged animals and associated with larger infarct size (11). All this highlights the need for large, multicenter biosampling studies in ICH, with standardized protocols and serial sampling including sampling weeks to months from ICH ictus (26).

Our study produced several additional novel findings. First was the association of the collagenases—MMP 1 and MMP 8–with clinicoradiologic deterioration. In particular, subacute levels of MMP 8 were positively correlated with absolute PHE in our cohort. These findings warrant further study in preclinical models of ICH and in ICH patients. In studies of bacterial meningitis, MMP 8 degrades occludin, an integral plasma-membrane protein present in the tight junctions that form the BBB (27, 28). And other studies have demonstrated a role for MMP 8 in modulating Th1/Th2 polarization and the production of TNFα by activated microglia (29–31).

The other novel finding of this study regards the potential use of these markers in diagnosing ICH due to CAA. Previous work has identified brain tissue changes in TGF-β, fibronectin, and MMP activation in CAA models (12, 32–36). For instance, in one study of aged transgenic mice, TGF-β1 was central to the deposition of beta amyloid in cerebral blood vessels and the meninges in response to tissue injury (12). There is far less literature examining the expression of these cytokines peripherally (e.g., in the serum) of CAA patients. Similar to the study by Greenberg et al. TGF-β levels alone were not diagnostic for CAA-related ICH (13). When evaluated in conjunction with fibronectin and our MMP panel, though, there was a unique marker signature in CAA ICH patients compared to hypertensive hemorrhage patients, in 2 independent study site cohorts. There is a need for a serum marker of CAA to complement clinical diagnostic criteria such as the MRI-based modified Boston criteria [especially as their sensitivity/specificity is decreased in patients who have yet to present with a symptomatic ICH, (37, 38)].

There are several limitations to this study. These are preliminary findings, and the size of our patient cohort is small. That being said, our patients were sampled in a serial fashion (essentially increasing our n) and the results demonstrated here could be considered hypothesis-generating. We did not find as many statistically significant associations of markers obtained during later timepoints, likely in part due to the fact that our sample numbers decreased if patients were discharged or died prior to day 10. We included patients with primarily moderate-sized, supratentorial hemorrhages only, thereby limiting the generalizability of our findings. Finally, regarding our ROC analysis, we excluded patients with lobar hemorrhages clinically determined to be due to causes other than CAA; that being said, there may be a subset of these patients who, upon obtaining tissue from hematoma evacuation or at autopsy, indeed have evidence of CAA.

## Ethics statement

This study was carried out in accordance with the recommendations of the Institutional Review Boards at the University of Texas Health Science Center at Houston and Hartford Hospital. The protocol was approved by the Institutional Review Boards at both institutions. All subjects gave written informed consent in accordance with the Declaration of Helsinki.

## Author contributions

MH: performing assays, organizing data, constructing initial manuscript drafts, editing drafts. LZ: all statistical analyses, editing drafts. LS: data acquisition/interpretation, editing drafts. NG: editing drafts. LM: study design, funding/sourcing assays, editing drafts. NE: study design, data acquisition/interpretation, writing the manuscript, editing drafts.

### Conflict of interest statement

The authors declare that the research was conducted in the absence of any commercial or financial relationships that could be construed as a potential conflict of interest.
